# Trimeric protein vaccine based on Beta variant elicits robust immune response against BA.4/5-included SARS-CoV-2 Omicron variants

**DOI:** 10.1186/s43556-023-00121-7

**Published:** 2023-03-10

**Authors:** Cai He, Li Chen, Jingyun Yang, Zimin Chen, Hong Lei, Weiqi Hong, Xiangrong Song, Li Yang, Jiong Li, Wei Wang, Guobo Shen, Guangwen Lu, Xiawei Wei

**Affiliations:** Laboratory of Aging Research and Cancer Drug Target, State Key Laboratory of Biotherapy and Cancer Center, National Clinical Research Center for Geriatrics, West China Hospital, Sichuan University, Chengdu, 610041 China

**Keywords:** RBD, Vaccine, Immune response, SARS-CoV-2, Omicron, Variants

## Abstract

The current Coronavirus Disease 2019 (COVID-19) pandemic, induced by newly emerging severe acute respiratory syndrome coronavirus 2 (SARS-CoV-2) Omicron variants, posed great threats to global public health security. There is an urgent need to design effective next‑generation vaccines against Omicron lineages. Here, we investigated the immunogenic capacity of the vaccine candidate based on the receptor binding domain (RBD). An RBD_β_-HR self-assembled trimer vaccine including RBD of Beta variant (containing K417, E484 and N501) and heptad repeat (HR) subunits was developed using an insect cell expression platform. Sera obtained from immunized mice effectively blocked RBD-human angiotensin-converting enzyme 2 (hACE2) binding for different viral variants, showing robust inhibitory activity. In addition, RBD_β_-HR/trimer vaccine durably exhibited high titers of specific binding antibodies and high levels of cross-protective neutralizing antibodies against newly emerging Omicron lineages, as well as other major variants including Alpha, Beta, and Delta. Consistently, the vaccine also promoted a broad and potent cellular immune response involving the participation of T follicular helper (Tfh) cells, germinal center (GC) B cells, activated T cells, effector memory T cells, and central memory T cells, which are critical facets of protective immunity. These results demonstrated that RBD_β_-HR/trimer vaccine candidates provided an attractive next-generation vaccine strategy against Omicron variants in the global effort to halt the spread of SARS-CoV-2.

## Introduction

COVID-19 is a respiratory infectious disease caused by SARS-CoV-2, which has evolved into a global pandemic and attracted extensive attention all over the world [1]. SARS-CoV-2 is a single-stranded enveloped RNA virus with a positive-sense genomic RNA of ∼30 kb that encodes four main constitutive proteins including spike, membrane, envelope, and nucleocapsid [2]. Spike (S) protein, compromising S1 and S2 functional subdomains, is located on the viral envelope, which is significant for fusion of the viral envelope with the host cell membrane [3]. S1 subunit contains RBD, which is primarily crucial for binding the virus to hACE2-expressing cells, while the S2 subunit typically consists of HR domains, comprising HR1 and HR2, which are strongly associated with the fusion of viral envelope and cellular membrane [4]. Since SARS-CoV-2 is continuously evolving, 227 vaccine candidates are under development, with 788 trials under clinical evaluation and 47 vaccines authorized for emergency use in human beings (by the end of August 2022) https://covid19.trackvaccines.org/.

Previously circulating variants of concern (VOCs), including Alpha, Beta, Gamma, and Delta, are de-escalated by public health agencies as the variants that are no longer circulating (https://outbreak.info/situation-reports). Meanwhile, the emergence of Omicron lineages has aroused deep concerns about the efficacy of existing vaccines. The Omicron variants were initially been divided into five subvariants including BA.1, BA.2, BA.3, BA.4, and BA.5 [5, 6], which share key mutations that have decreased antibody neutralization against current vaccines [7–10]. BA.1 was the dominant Omicron mutant in the initial wave and was subsequently replaced by BA.2. BA.3 lineage was an integration of mutations in BA.1 and BA.2 S proteins. In terms of mutations, BA.4 and BA.5 differ from each other in mutations outside the S gene sequences, but they are identical in S protein. Most remarkably, Omicron BA.4/5 lineages appear more resistant to previous SARS-CoV-2 vaccines compared with BA.1 and BA.2 [5, 6]. Two-dose Pfizer-BioNTech vaccine offered an obvious decline in neutralizing antibody titers [11]; single dose of Ad26.COV2.S, two doses of Sputnik V, two doses of BBIBP-CorV [12], and triple doses of AstraZeneca or Pfizer vaccine [5] all showed negligible neutralization against Omicron variants; Two doses of BNT162b2 and AZD1222 vaccine showed rarely no neutralizing antibodies after 150 days [13]. A series of current studies suggested that some previous vaccines have reduced efficacy against the newly predominant Omicron variants and developing specific vaccines against Omicron variants is urgently needed.

Omicron variants are characterized by a large number of mutations with 26 to 32 changes in regions of S protein, which is recognized by specific antibodies, damping vaccine potency [14]. Notably, the Omicron variant shares the same three significant mutation sites as the previous VOC Beta variant, including K417, E484, and N501, which provides constructive guidance to design a highly effective vaccine against recently emerged Omicron. Tubiana et al. investigated the level of cross-reactive antibodies in Beta-immunized sera, and the results demonstrated that Beta sera displayed higher cross-reactivity with BA.1 (49%) than WT sera (31%) [15], suggesting Beta immunized sera seemed to be more effective against Omicron variants. On the one hand, RBD is the major antigenic target of SARS-CoV-2 vaccine design, but monomeric RBD lacks potent immunogenicity on account of its small molecular size for antigen presentation by antigen-presenting cells [16]. Numerous engineered-RBD dimeric or trimeric constructs have emerged to improve the immunogenicity of RBD [17]. On the other hand, HR1 and HR2 regions are conserved according to different SARS-CoV-2 strains, which can self-assemble to form a 6-helix bundle structure, also known as trimers of HR1-HR2 [18].

In the present study, we constructed a trimer RBD_β_-HR protein by the baculovirus-insect cell expression system, in which the RBD sequences within SARS-CoV-2 sharing Beta variant (K417, E484, N501) fused with HR1 and HR2 regions to form a trimeric structure. The RBD_β_-HR/trimer vaccine not only elicited robust a humoral immune response against SARS-CoV-2 mutant strains, especially Omicron variants but also induced a potent cellular immune response, which is of great significance for containing the spread of SARS-CoV-2.

## Results

### Characterization of RBD_β_-HR/trimer protein

**Fig. 1:**
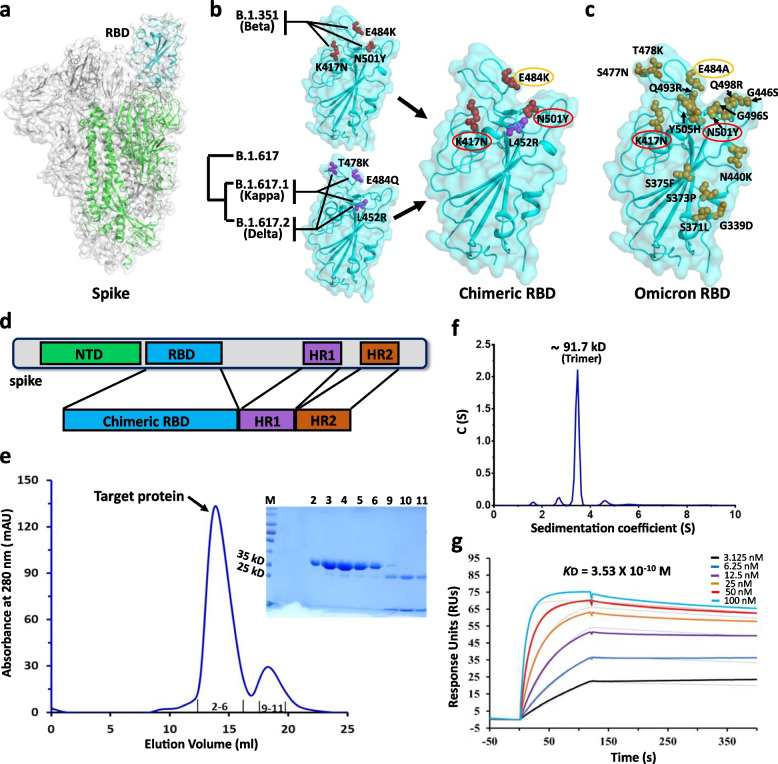
Antigen design and RBD_β_-HR/trimer characterization. **a** An overview of the structure of SARS-CoV-2 spike trimer. RBD is a key component in vaccine development, which is highlighted and marked in Cyan. **b** An overview of our chimeric RBD design. Beta-specific substitutions of K417N, E484K and N501Y, as well as the amino acid mutation of L452R shared by the Kappa and Delta VOCs, are included to yield the final RBD design. **c** An overview of the residue substitutions identified in Omicron spike RBD. The K417N and N501Y mutations in our design, which are also shared by Omicron, are highlighted by encircling with red lines. The substitution of E484, by a lysine in our design but by an alanine in Omicron, is marked with yellow circles. **d** The chimeric RBD is further fused with SARS-CoV-2 spike HR1 and HR2 to yield the final antigen design. **e** The recorded solution behavior of the target protein on a Superdex 200 Increase column. The elution chromatograph of the protein and the SDS-PAGE analyses of the pooled samples are shown. **f** Characterization of RBD_β_-HR trimer with analytical ultra-centrifugation (AUC). The recorded sedimentation profiles are shown. **g** Characterization of the interaction between our target protein and ACE2 with surface plasmon resonance (SPR). The real-time binding profiles are shown

RBD is a crucial target in the design of SARS-CoV-2 vaccines (Fig. 1a). Beta variant contains multiple mutations in the S protein, including a trio of mutation sites (K417N, E484K and N501Y) in RBD, and Kappa variant and Delta variant share the amino acid mutation L452R, which is included to obtain the chimeric RBD. Meanwhile, the mutations of RBD in Omicron variants (Fig. 1b-c) have in common with Kappa, Delta, Alpha, Beta and Gamma variants, which are particularly noteworthy mutations [19]. Based on this, the study designed a trimeric RBD_β_-HR protein in which the chimeric RBD was further fused with HR1 and HR2 regions, which was produced in insect cells infected with recombinant baculoviruses containing the RBD_β_-HR sequence. Within the constructed protein, chimeric RBD belongs to S1 subunit, and HR1-HR2 six-helix bundle belongs to S2 subunit, making the RBD_β_-HR protein considerably self-assemble to form a native-like trimeric structure (Fig. 1d). After purification, a peak of RBD_β_-HR/trimer protein was detected by size exclusion chromatography and polyacrylamide gel electrophoresis (SDS-PAGE) that proved the formation of trimers (Fig. 1e). The molecular weight was further corroborated as 91.7 kD by analytical ultra-centrifugation (AUC) (Fig. 1f) and the binding affinity was calculated to be about *K*_*D*_ = 3.53 × 10^–10^ M (dissociation constant, Fig. 1g).

### RBD_β_-HR/trimer vaccine elicited a potent humoral immune response

**Fig. 2:**
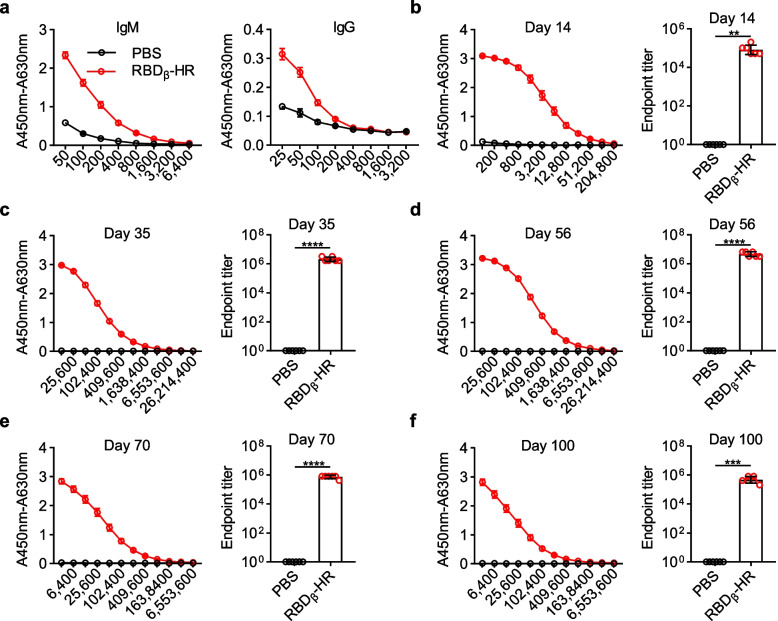
RBD specific binding antibody in immunized mice. Mice were immunized with 10 μg RBD_β_-HR/trimer protein per mouse in 100 μL in the presence of MF59-like adjuvant with prime and booster vaccinations at an interval of 21 days, compared with the control group that received PBS with a volume of 100 μL. **a** Sera were obtained from the mice 7 days after the prime vaccination and the levels of IgG and IgM against RBD protein were detected at a serial of serum dilutions. **b-f** The levels of IgG at a serial of serum dilutions (left) and the endpoint titers (right) of the sera obtained on day 14 (**b**), 35 (**c**), 56 (**d**), 70 (**e**), 100 (**f**) after the prime vaccination. The absorbance at 450 nm was measured using a microplate reader with the wavelength correction set to 630 nm. Data are geometric mean ± SD. *p* value < 0.05 were considered statistically significant (*p < 0.05; **p < 0.01; ***p < 0.001; ****p < 0.0001)

Focusing on Omicron, we designed a trimeric RBD protein containing the key mutation sites in both Beta and Omicron variants. MF59-like adjuvant has been integrated to formulate the RBD_β_-HR/trimer vaccine. Mice (*n* = 6 per group) were intramuscularly injected with 10 μg RBD_β_-HR/trimer protein with MF59-like adjuvant on days 0, 21, and 42, and mouse sera were collected 7 or 14 days after each immunization. To evaluate the immunogenicity of RBD_β_-HR/trimer vaccine, an enzyme-linked immunosorbent assay (ELISA) was used to determine RBD-specific antibody titers. Sera obtained on day 7 after the prime dose of RBD_β_-HR/trimer vaccine exhibited apparent RBD-specific IgM and IgG antibody responses to RBD protein (Fig. 2a). At the early stage, RBD_β_-HR/trimer vaccine showed a higher level of RBD-specific IgM than IgG antibody titers (Fig. 2a). Subsequently, a much higher level of RBD-specific IgG antibody titers was observed on day 14 than that of day 7 (Fig. 2b). The specific IgG antibody titers were increased significantly after the first booster vaccination, while they were elevated gently after the second booster immunization (Fig. 2c-d). The geometric mean titers (GMTs) of RBD-specific IgG antibodies on day 14, day 35, and day 56 were 8.1 × 10^4^, 2.1 × 10^6^, and 4.6 × 10^6^ respectively, and GMTs on day 28 increased by 25-fold and on day 56 by 48-fold compared with GMTs on day 14. After the second booster vaccination, the level of RBD-specific IgG antibodies maintained high for at least 100 days (Fig. 2e-f). The results suggested that the RBD_β_-HR/trimer vaccine could induce potent RBD-specific antibody responses, which indicated that the RBD_β_-HR/trimer vaccine had strong immunogenicity.

### The RBD_β_-HR/trimer vaccine elicited strong antibodies to inhibit the binding of RBD (Omicron) to hACE2

**Fig. 3:**
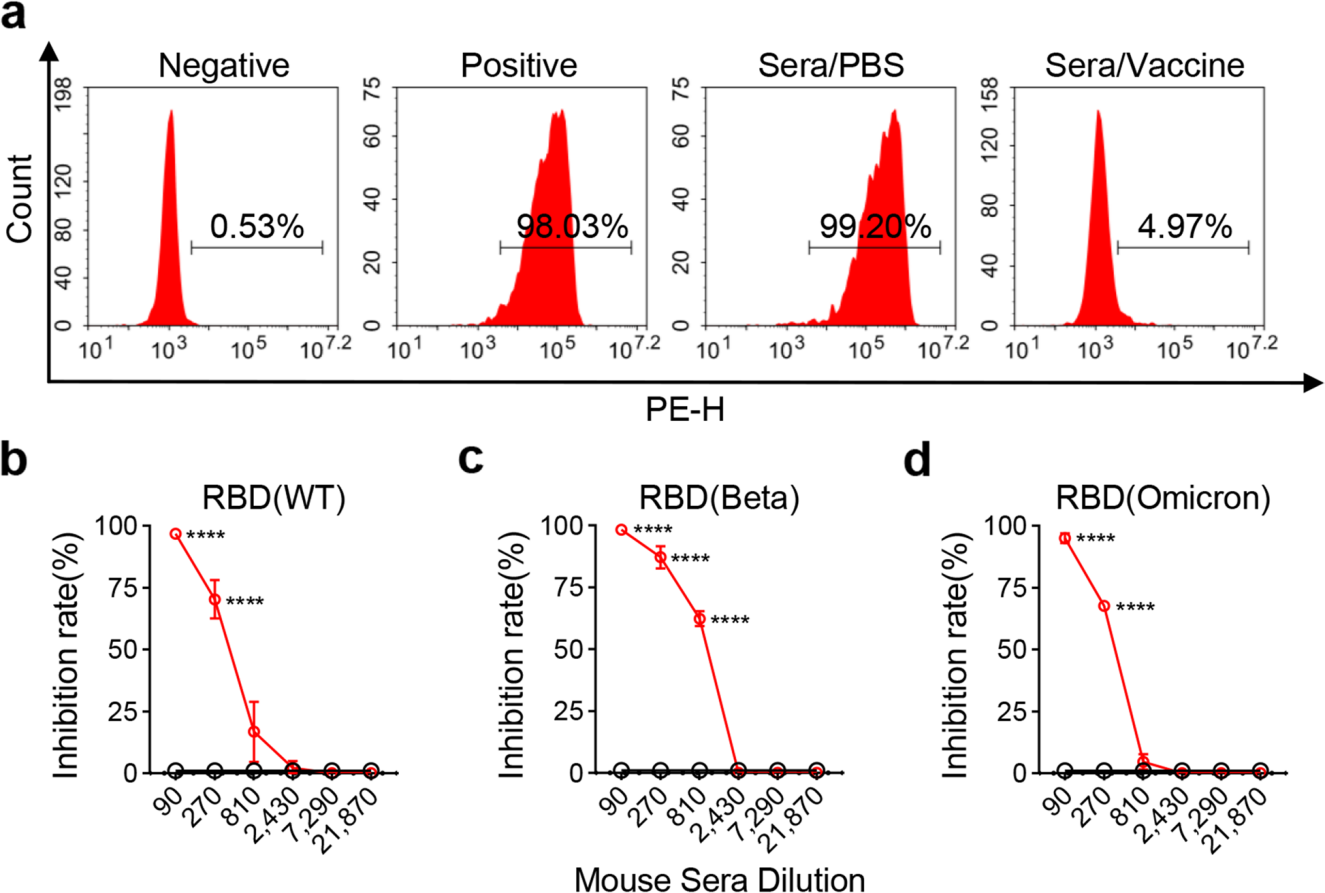
Inhibition of the binding of the RBD to hACE2 receptor. **a** SARS-CoV-2 RBD (Omicron)-Fc fusion protein was added to 293 T/hACE2 cells in the presence or absence of sera at a dilution of 1:90, followed by incubation with anti-human IgG-PE labeled antibodies. The representative graphs followed the treatment from the left to right orderly: cells were stained with anti-human IgG-FITC conjugated antibody alone in the absence of RBD-Fc fusion protein (negative control); the cells were incubated with RBD-Fc fusion protein pre-incubated in the absence of sera (positive control); the cells treated with the sera from PBS group (sera/PBS); the cells treated with the sera from the vaccine group (sera/vaccine). **b-d** Inhibition rate of RBD-WT (**b**), RBD (Beta) (**c**), RBD (Omicron) (**d**) binding to cell surface ACE2 receptor in the presence of sera obtained day 56. Data are mean ± SEM. *p* value < 0.05 were considered statistically significant (*p < 0.05; **p < 0.01; ***p < 0.001; ****p < 0.0001)

Immune sera obtained from mice were detected for the blocking activity of RBD-hACE2 binding. Three RBD-Fc fusion proteins: RBD (WT), RBD (Beta), and RBD (Omicron) were utilized to bind hACE2. In the absence of serum, RBD (Omicron)-hACE2 positivity was detected in 98.03% of 293 T/hACE2 cells (293 T cells stably expressing hACE2) as a positive control; without RBD (Omicron)-Fc fusion protein, 0.53% of the cells were positively detected as a negative control (Fig. 3a). Immune sera from PBS group exhibited no or less inhibitory effects with 99.20% RBD-hACE2 positivity in 293 T/hACE2 cells (Fig. 3a). On the contrary, only 4.97% of 293 T/hACE2 cells were positive for RBD (Omicron)-hACE2 (Fig. 3a). Based on the excellent blocking effect of the vaccine, we further compared the blocking efficacy of the sera on the binding of 293 T/hACE2 cells to three RBD proteins, including RBD (WT), RBD (Beta), and RBD (Omicron). Remarkably, with the sera obtained from RBD_β_-HR/trimer vaccine treated mice, it enabled the sera block RBD (WT) binding to 293 T/hACE2 cells (Fig. 3b), and showed superior ability to block RBD (Beta) (Fig. 3c), and exhibited efficacy in blocking RBD (Omicron) to some extent (Fig. 3d). Although the RBD_β_-HR/trimer vaccine showed effective blockade against Omicron variant, the vaccine performed well at blocking Beta variant. The results suggested that RBD_β_-HR/trimer vaccine not only retained efficacy against prototype SARS-CoV-2, but also found to be effective against major variant Omicron.

### Susceptibility of SARS-CoV-2 Omicron variants to neutralization

**Fig. 4:**
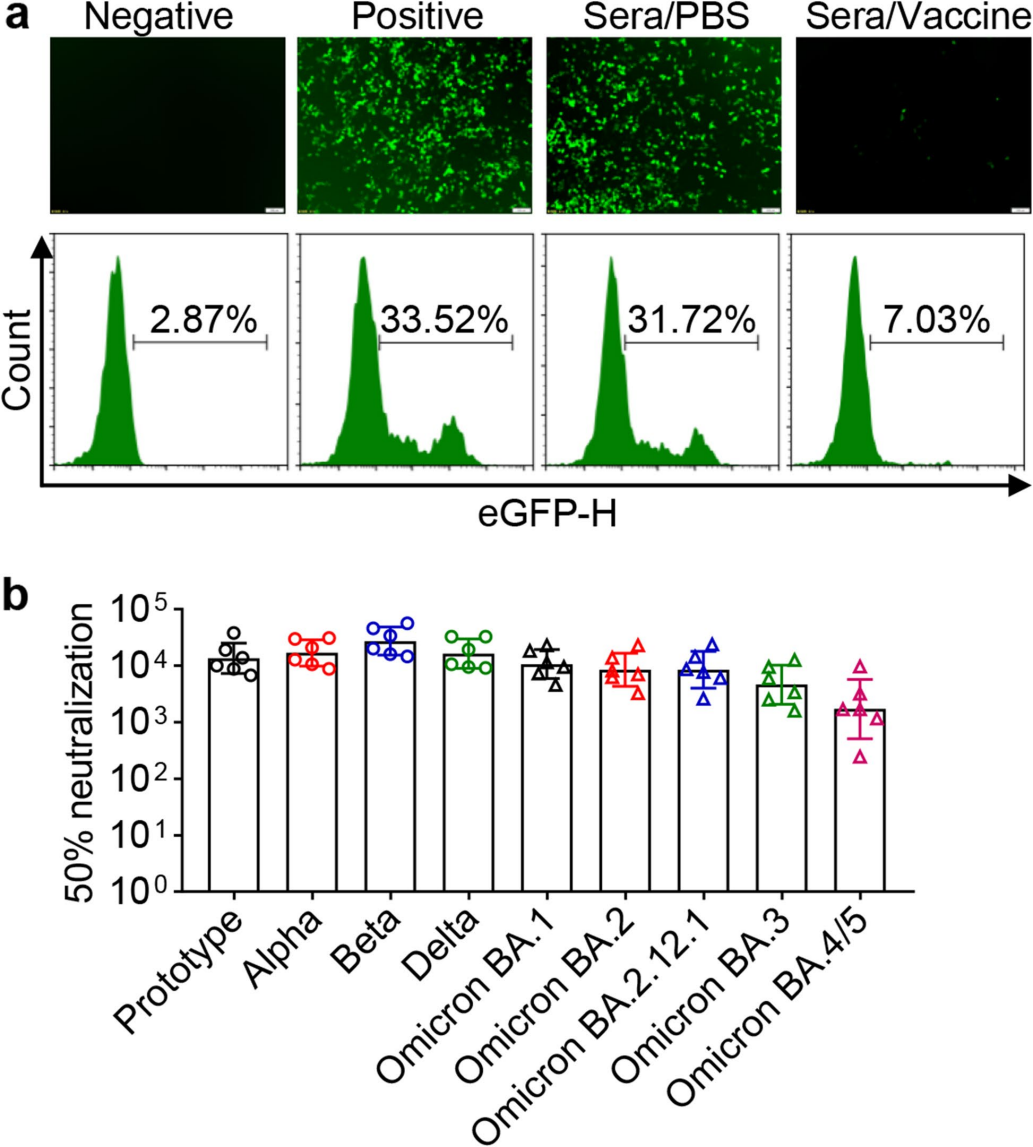
Neutralization of SARS-CoV-2 pseudovirus infection in 293 T/hACE2 cells by immune sera. **a** Representative graphs followed the treatment from the left to right orderly: infection without SARS-CoV-2 pseudovirus (negative control); infection with SARS-CoV-2 pseudovirus without sera (positive control); sera from mice treated with PBS (sera/PBS); sera obtained from mice on day 56 treated with the vaccine at 1:90 dilution (sera/vaccine). **b** Neutralizing antibody titers of the immunized sera on day 56 against the pseudoviruses bearing either prototype, Alpha, Beta, Delta, Omicron lineages BA.1, BA.2, BA.2.12.1, BA.3, BA.4/5. Data are geometric mean ± SD

Assessment of neutralizing activity against prototype and several variants of concern is beneficial to determine whether the vaccine is effective [20]. We evaluated the neutralizing activity of the sera obtained from the mice, immunized with RBD_β_-HR/trimer vaccine, against SARS-CoV-2 pseudoviruses (EGFP-Luciferase) within 293 T/hACE2 cells. EGFP-expressing cells drastically diminished when Omicron (BA.1) pseudovirus was pre-incubated in the sera from mice intramuscularly injected with RBD_β_-HR/trimer vaccine at a 1:90 dilution (Fig. 4a).

To further investigate the neutralization efficacy of the immune sera against wildtype and mutant SARS-CoV-2 variants. We performed pseudoviruses neutralization assay containing prototype, Alpha, Beta, Delta, and Omicron lineages BA.1, BA.2, BA.2.12.1, BA.3, and BA.4/5 pseudoviruses. The Beta pseudovirus was the most related to RBD_β_-HR/trimer among the variants. The vaccine elicits the highest neutralizing antibodies in mice against the Beta variant with the GMTs of 27,338 for 50% neutralization (Fig. 4b). Furthermore, the GMTs of 50% neutralization against the wildtype, Alpha, and Delta variants were 13,480, 16,885, and 16,409, respectively (Fig. 4b). There were different degrees of the decline of neutralization antibodies against the Omicron lineages in comparison to prototype variant. The neutralizing antibody titers of the immune sera against the Omicron BA.1 variant were only decreased by 1.26-fold and 2.55-fold compared with prototype and Beta pseudoviruses, respectively. The GMTs of 50% neutralization against Omicron BA.2 (8488) corresponded to subvariant BA.2.12.1 (8469), which were 1.59-fold lower than the prototype pseudovirus. Although BA.3 and BA.4/5 showed the ability to evade immunity from the vaccine compared with other Omicron lineages, the GMTs of 50% neutralization still reached 4641 and 1716, respectively (Fig. 4b). RBD_β_-HR/trimer vaccine could induce cross-protection against SARS-CoV-2 VOCs and significantly improve the neutralization capacity against Omicron variants.

### RBD_β_-HR/trimer vaccine elicited a robust T cell response

**Fig. 5:**
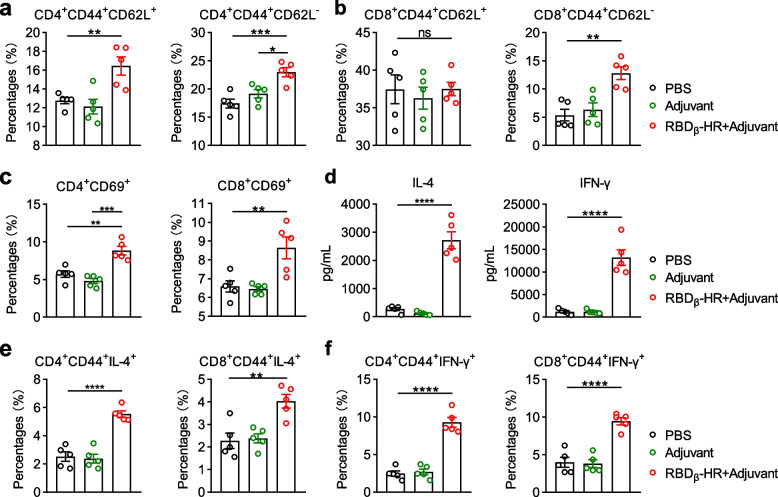
RBD_β_-HR/trimer vaccine elicits robust cellular immune response. Mice immunized with PBS, MF59-like adjuvant, and RBD_β_-HR/trimer vaccine were sacrificed on day 49. **a** The percentages of central memory CD4^+^ T cells (left) and effector memory CD4^+^ T cells (right) in the spleens. **b** The percentages of central memory CD8^+^ T cells (left) and effector memory CD8^+^ T cells (right) in the spleens. **c** The percentages of activated CD4^+^ T cells (left) and activated CD8^+^ T cells (right) in the spleens. **d** Cytokines secreted by the spleen lymphocytes into the supernatants after stimulation with RBD_β_-HR/trimer protein for 72 h. **e** The percentages of memory CD4^+^ T cells (left) and memory CD8^+^ T cells (right) secreted IL-4 in the spleen lymphocytes after stimulation with RBD_β_-HR protein for 72 h. **f** The percentages of memory CD4^+^ T cells (left) and memory CD8^+^ T cells (right) secreted IFN-γ in the spleen lymphocytes after stimulation with RBD_β_-HR/trimer protein for 72 h. Data are mean ± SEM. *p* value < 0.05 were considered statistically significant (*p < 0.05; **p < 0.01; ***p < 0.001; ****p < 0.0001)

To determine the ability of RBD_β_-HR/trimer vaccine to generate a T cell response, mice (*n* = 5 per group) were immunized with PBS, MF59-like adjuvant, RBD_β_-HR/trimer adjuvanted with MF59-like adjuvant in a three-dose spaced 21 days apart and euthanized on day 49. It is well known that memory T cells may be CD4^+^ or CD8^+^ and are divided into effector memory (CD44^+^ CD62L^−^) and central memory (CD44^+^ CD62L^+^) populations by function and phenotype [21]. Effector memory T cells can be preferentially attracted to sites of inflammation and rapidly produce IFN-γ or IL-4 upon restimulation, while central memory T cells promote their migration through the lymph nodes that enhance their ability to interact with antigen-presenting cells [22]. In spleen samples, the percentages of central memory and effector memory T cells in CD4^+^ T cell populations drastically increased in RBD_β_-HR/trimer vaccine group compared with the control groups (Fig. 5a). However, the percentages of effector memory T cells were obviously elevated, while central memory T cells remained unchanged in CD8^+^ T cell populations (Fig. 5b). In addition, activated CD4^+^ CD69^+^ and CD8^+^ CD69^+^ T cells were markedly increased compared with those in the control groups (Fig. 5c). To further investigate the role of RBD_β_-HR/trimer protein in cellular immunity, lymphocytes isolated from the spleen were re-stimulated with RBD_β_-HR/trimer protein for 72 h. After ex vivo stimulation, RBD_β_-HR/trimer protein facilitated the simultaneous release of IL-4 and IFN-γ by lymphocytes (Fig. 5d). Importantly, the proportion of IL-4^+^ and IFN-γ^+^ cytokine-secreting CD4^+^ CD44^+^ and CD8^+^ CD44^+^ effector memory T cells was significantly higher with RBD_β_-HR/trimer stimulation (Fig. 5e-f).

### RBD_β_-HR/trimer vaccine promoted Tfh cell and GC B cell generation

**Fig. 6:**
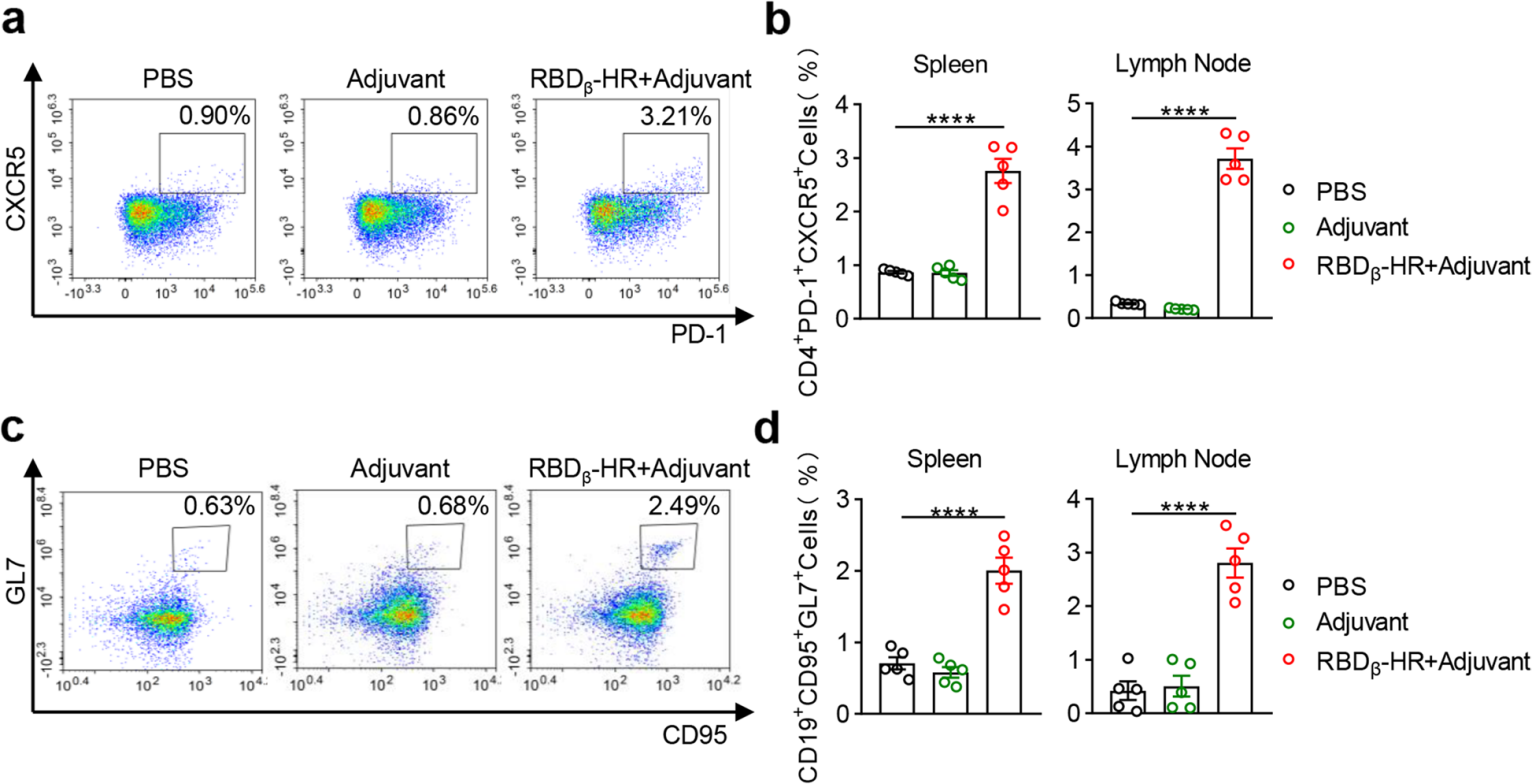
RBD_β_-HR/trimer vaccine promotes the generation of Tfh cells and GC B cells. Mice immunized with PBS, MF59-like adjuvant, and RBD_β_-HR/trimer vaccine were sacrificed on day 49. **a** Representative graphs of Tfh cells marked as CD4^+^ CXCR5^+^ PD-1^+^. **b** The percentages of Tfh cells in the spleens and draining lymph nodes. **c** Representative graphs of GC B cells marked as CD3^−^ CD19^+^ GL7^+^ CD95^+^. **d** The percentages of GC B cells in the spleens and draining lymph nodes. Data are mean ± SEM. *p* value < 0.05 were considered statistically significant (*p < 0.05; **p < 0.01; ***p < 0.001; ****p < 0.0001)

As demonstrated before, vaccination with RBD_β_-HR/trimer vaccine triggered a considerable RBD specific humoral response and antigen-specific T cell response. Basically, the formation of GC in secondary lymphoid organs, in which specific antibody-producing plasma cells and memory B cells are rapidly generated, is a major part of the adaptive immune response to vaccines [23, 24]. During the GC reaction, Tfh cells are pivotal providers of T cells that help B cells by releasing cytokine and costimulatory signals [25], while GC B cells contribute to affinity-matured memory B cells and long-lived plasma cells [26]. We next investigated the efficacy of the candidate vaccine on GC formation by observing the percentages of CD4^+^ Tfh cells (CD4^+^ CXCR5^+^ PD-1^+^) and GC B cells (CD3^−^ CD19^+^ GL7^+^ CD95^+^) [27] in both spleen and lymph node samples 7 days after the final immunization. Robust Tfh cells were found in the spleen and draining lymph node after three doses of RBD_β_-HR/trimer vaccine in immunized mice (Fig. 6a-b). In line with the Tfh cells, the percentages of GC B cells were dramatically elevated in the spleen and draining lymph node compared with PBS and adjuvant groups (Fig. 6c-d). The findings showed that GC formation following immunization with RBD_β_-HR/trimer vaccine was substantially increased, which confirmed the ability of the vaccine to elicit robust humoral immunity.

## Discussion

Omicron variants with key mutations in S protein have led to the major pandemic and impaired efficacy of vaccines worldwide [28]. Based on the key mutation sites sharing similarly in Beta and Omicron variants, we designed RBD_β_-HR/trimer protein to provide a promising vaccine candidate against Omicron lineage variants. RBD_β_-HR/trimer vaccine induced robust and durable humoral immunity and cellular immunity. The immunized mice exhibited rapidly and durably high RBD_β_-HR/trimer-specific binding antibody titers. Further neutralization assay confirmed that the vaccine-elicited neutralizing antibodies prominently blocked RBD-mediated hACE2 binding and reduced the entry of various SARS-CoV-2 pseudoviruses into 293 T/hACE2 cells. The neutralization antibody elicited by RBD_β_-HR/trimer vaccine not only performed well against Omicron and its lineages, but also had a good effect on pseudoviruses of prototype SARS-CoV-2, Alpha, Beta, and Delta variants. Furthermore, RBD_β_-HR/trimer vaccine induced robust cellular responses in both the spleens and lymph nodes of immunized mice compared with the control groups.

HR region in the S2 subunit of S protein was included in the design of RBD_β_-HR/trimer protein. As reported before, RBD protein induced a weak immune response due to its poor immunogenicity and RBD-only vaccines failed to elicit long-lasting protective immunity [16].Intriguingly, HR was highly conserved in the evolutionary history of β-coronaviruses and held the potential to induce neutralization activity [18, 29]. To increase the immunogenicity of the protein subunit vaccine, our RBD_β_-HR/trimer antigen design was based on the rationale that the HR subdomain can be automatically assembled into a six-helix bundle structure. We directly connected the RBD subunits of the beta variant with the HR subdomain formed to the trimeric protein, which could trigger strong immune response against Omicron variants and maintain a stable natural trimer conformation. Several studies have shown that polymer protein vaccines may elicit more desirable immune responses than vaccines comprised of monomeric antigens [18, 30, 31]. Notably, RBD_β_-HR/trimer protein was constructed by the baculovirus-insect cell expression system, which was extensively utilized to yield RBD [32], glycosylated S1 domain [33], S protein [34], SARS-CoV-2 virus-like particles [35], and so on. As a typical eukaryotic expression system, the baculovirus-insect cell expression system provides post-translational modifications that affect the immunogenicity of the recombinant protein and determine the immunological protection and valence of vaccines [36]. Besides, the recombinant proteins expressed by the baculovirus-insect cell expression system are well-folded and soluble, and include the desired post-translational modifications [37], which not only induce robust immune responses in organisms, but also present the interest antigen domain in the self-assembling structure rapidly, providing an accessible strategy to obtain a safe, effective, scalable, and affordable vaccine candidate for newly emerging SARS-CoV-2 variants.

Antigen-specific antibodies have been generally considered as conferring vaccine-induced protection against SARS-CoV-2 [38]. IgM, the earliest immunoglobin, is secreted by the adaptive immune system 5–7 days in response to a foreign antigen. For vaccine design and evaluation, a high prevalence of IgM antibodies versus the other antibodies shows a rapid response to immunogenicity of antigen [39]. In our study, the mice immunized with RBD_β_-HR/trimer vaccine elicits robust RBD-specific IgM and slight IgG responses 7 days after the prime immunization, which underwent a significant isotype switching to produce increasingly stronger IgG responses following subsequent boosting immunization. After completion of the immunization, the GMTs of RBD IgG is approximately 1 × 10^6^, and the results are consistent with mice immunized with 10 μg of RBD mixed with alum or soluble CpG or AMP-CpG [40], and 5 μg of RBD mixed with alum [32], demonstrating that the vaccines can elicit a rapid and durable humoral immune response. Furthermore, IgG titers generally have a close relationship with neutralizing titers, and quantitation of neutralization potency is an indicator of immune protection of SARS-CoV-2 vaccines from SARS-CoV-2 variants [41]. In this context, the sera from immunized mice protected the 293 T/hACE2 cells from infection with pseudovirus (Omicron BA.1), with PVNT_50_ GMTs of 10,727, which was only 1.26-fold and 2.55-fold lower compared to the pseudovirus (prototype) and pseudovirus (Beta). RBD_β_-HR/trimer vaccine remained effective against Omicron lineages with a slight but significant decrease in neutralization that was more apparent in BA.3 and BA.4/5. Several studies have also examined the ability of immunized sera against Omicron lineages. In July 2022, a new study demonstrated that the neutralizing titer of BA.4/5 by a three-dose vaccine (AZD1222 and BNT162b2) is decreased by 1.8 ~ 3.1-fold in comparison to BA.1 and BA.2 [5]. The sera obtained from recipients vaccinated with mRNA-1273 vaccine were tested, and it was found that Omicron was 41~84 folds less sensitive to neutralization than D614G and 5.3~7.4 folds less sensitive than Beta [42]. A two-dose mRNA vaccine or a single-dose J&J vaccine are modestly effective against Omicron variant [43]. Although these immune sera had slightly reduced but overall widely preserved neutralization titers against Omicron lineage pseudoviruses. All the data demonstrate that RBD_β_-HR/trimer vaccine could induce broader cross-protective neutralizing antibodies against SARS-CoV-2 variants, especially Omicron lineages.

The cellular immune responses underpin vaccine efficacy and can be related to the clearance of SARS-CoV-2 infection that CD4^+^ and CD8^+^ T cells participate [44]. CD4^+^ T cells promote antibody maturation, while CD8^+^ T cells are crucial for killing virus-infected cells. Using a strategy of sampling immunologically relevant tissues to vaccination, we showed that the vaccine mobilized the cellular drivers of a multifactorial immune response. In spite of no significant differences in central memory CD8^+^ T cells at day 49 postvaccination, we did observe significant differences in both CD4^+^ and CD8^+^ T cells in terms of effector memory cells and activated cells. It was demonstrated that the diversified cellular responses, involving CD4^+^ T cell and CD8^+^ T cell activation and durable effector-memory functional CD4^+^ and CD8^+^ T cell responses at secondary lymphoid organs, are related to blocking the entry of viruses [45]. With the stimulation of RBD_β_-HR/trimer protein, the levels of IL-4 and IFN-γ were elevated from the supernatant in splenic lymphocyte proliferation, of which the level of IFN-γ was more elevated. Similarly, the proportion of cytokine-secreting lymphocytes was greater in the IFN-γ-specific memory CD4^+^ and CD8^+^ T cell population than in the IL-4-specific memory CD4^+^ and CD8^+^ T cell populations. It was suggested that RBD_β_-HR/trimer vaccine adjuvanted with adjuvant mobilized both Th1 cells (producing IFN-γ) and Th2 cells (producing IL-4) to participate in the immune response, showing a mixed Th1/Th2 cellular response whereas a Th1 bias [46]. Importantly, a sizable CD4^+^ Tfh cell population and GC B cell population were present in the spleen, and similar findings emerged from the analysis of draining lymph nodes of the RBD_β_-HR/trimer vaccine-immunized mice. As demonstrated previously, Tfh cells were a critical component of potent humoral immune responses [47], while GC B cells were strongly associated with the production of nAbs [48]. Given the multiple potential mediators of protection induced by RBD_β_-HR/trimer vaccine, the efficacy of the vaccine could be possibly preserved over a longer time, even with a substantial reduction in neutralization by immune sera.

In summary, a self-assembling RBD_β_-HR/trimer vaccine displaying the Beta-mutated RBD of SARS-CoV-2 adjuvanted with an MF59-like adjuvant exhibited robust humoral and cellular immune responses to Omicron and the other variants. In light of the significant immunogenicity and accessibility, the RBD-based recombinant protein subunit vaccine candidate exhibits a specific antigen domain in a self-assembling manner for platform construction and clinical trial translation.

## Materials and methods

### Cell culture and animals

HEK293T cells were acquired from the American Type Culture Collection (ATCC). 293 T/hACE2 cell was engineered by transduction of hACE2 into HEK293T cell (293 T/hACE2 cell), followed by stable cell selection as previously described [32]. 293 T cells and 293 T/hACE2 cells were cultured in DMEM (4.5 g/L glucose, Gibco) enriched with standardized 10% FBS (PAN) and 1% penicillin–streptomycin (Gibco) at 37 °C with 5% CO_2_.

8 weeks old female NIH mice were ordered from Vital River Laboratories (Beijing, China). All mice were fed in Animal Center at the School of Public Heath (Sichuan University, Chengdu, China) under specific pathogen-free (SPF) conditions. All mice were housed in conditions of a 24 h light–dark cycle and fed adaptively for 1 week with free access to feed and water.

### RBD_β_-HR/trimer protein expression and purification

The baculovirus-insect cell expression system (Invitrogen) was utilized to express the RBD of the S protein of SARS-CoV-2 Beta variant as previously described [32]. The RBD_β_-HR/trimer recombinant protein is constructed by the SARS-CoV-2 Beta and Delta variant RBD (amino acids 320–545) of K417N, E484K, N501Y, and L452R mutants and two heptapeptide repeats (HR1, amino acids 916–966; HR2, amino acids 1157–1203) of the SARS-CoV-2 Wuhan-Hu-1 isolate. The gene sequence of GP67-Trx-His-EK-RBD_β_-HR was transferred into the pFastBac1 vector, and then the bacmid was transfected into insect Sf9 cells by utilizing LipoInsect Transfection Regent (Beyotime, China). The cell culture supernatant containing the packaged recombinant baculovirus, was collected after 3 days. The baculovirus was expanded in Sf9 cells within 2 ~ 3 generations before being used for RBD_β_-HR/trimer protein harvest. For primary purification, the supernatants were collected and purified by a 5-mL HisTrap excel column (GE Healthcare), further purified on Superdex 200 Increase 10/300 GL columns (GE Healthcare), and ultimately dissolved in soluble protein buffer containing 20 mM Tris–HCl and 150 mM NaCl. The prepared RBD_β_-HR/trimer protein was investigated by SDS-PAGE and visualized with Coomassie blue.

### Vaccination and sera collection

MF59-like adjuvant was performed according to a previous report [49]. The MF59-like adjuvant was an oil-in-water emulsion consisting of squalene (4.3%) in citric acid buffer, Tween 80 (0.5%) and Span 85 (0.5%), which had similar manufacture craft and physicochemical properties to MF59. The RBD_β_-HR/trimer protein was mixed with MF59-like adjuvant at a volume ratio of 1:1. Six or five mice were enrolled in three groups at random. Mice received immunization by intramuscular injection on days 0, 21, and 42. Each group of mice was immunized with 100 µL volume of PBS, MF59-like adjuvant, or RBD_β_-HR/trimer vaccine (mixed equal volume of MF59-like adjuvant and RBD_β_-HR/trimer protein, 10 μg per mice). Pre-immunization blood sample was collected via the ocular veniplex before the prime vaccination and was collected on day 7 and/or day 14 after each vaccination. After standing for 2 h, sera were obtained by centrifuging at 6000 rpm and kept at -80 °C before use.

### Enzyme-linked immunosorbent assays

RBD protein was pre-coated into flat-bottom 96-well high binding plates at 100 ng per well in carbonate coating buffer and incubated overnight at 4 °C. After washing with PBS containing 0.05% Tween 20 (PBST), the background was blocked with a blocking buffer (PBST containing 1% bovine serum albumin, BSA) at 37 °C for 1 h. The mouse sera were serially diluted twofold in 1% BSA and then reacted with the RBD-coated wells at 25 °C for 1 h. Horseradish peroxidase (HRP)-conjugated rabbit anti-mouse IgG or IgM antibodies (Southern Biotech, USA) served as the secondary antibody at a 1:5,000 dilution for 1 h. Subsequently, washing with PBST five times, 100 µL/well of 3,3’,5,5’-tetramethyl biphenyl diamine (TMB) substrate (Thermo Fisher Scientific, USA) was added to the plates, then the reaction was quenched with 100 μL/well of stopping solution (Beyotime, China), and the optical density (OD) value was detected at 450 nm (630 nm as a reference) on a microplate reader. Titers were determined at an absorbance cutoff of 0.105 OD value.

Inflammatory cytokines secreted by spleen lymphocytes in the supernatants were detected by ELISA after incubating with RBD protein. Supernatant samples were harvested to measure the amount of IFN-γ and IL-4 by ELISA kits for mouse IFN-γ and IL-4 (Thermo Fisher Scientific, USA) following the manufacturer’s instructions. Cytokines concentration was determined by regression analysis utilizing a standard curve with recombinant cytokines.

### Blockade of RBD-Fc binding to receptor hACE2

hACE2 binding of RBD or mutated RBD was carried out by flow cytometry (FCM) as previously reported [32]. The sera were obtained from mice intramuscularly injected with RBD_β_-HR/trimer vaccine on day 56, and sera from mice administrated with PBS were used as a control. 400 ng/mL RBD (WT)-Fc, RBD (Beta)-Fc, and RBD (Omicron)-Fc proteins were incubated with a series of diluted mouse sera for half an hour at 37 °C prior to infection of 293 T/hACE2 cells. After incubation, the cells were cleaned with PBS and stained with PE-conjugated anti-human IgG Fc secondary antibody at 4 °C for an additional half an hour. The mean fluorescent intensity (MFI) was determined by a NovoCyte Flow Cytometer (ACEA Biosciences), and the data were managed by NovoExpress software.

### Pseudovirus neutralization assay

Pseudoviruses (EGFP-Luciferase) expressing the prototype SARS-CoV-2, Alpha, Beta, Delta, and Omicron lineage variants were obtained from Genomeditech (Shanghai, China). The above pseudoviruses were pre-incubated with serially diluted immune sera in 96-well plates for 1 h at 37 °C, and then 293 T/hACE2 cells (1.2 × 10^4^ per well) were added to the plates and incubated for 48 h. EGFP-expressing pseudovirus in 293 T/hACE2 cells was detected by fluorescence microscopy or FCM. To visualize the luciferase-expressing pseudovirus infection in 293 T/hACE2 cells, the supernatants were removed, followed by adding luciferase substrate (Promega, USA) to react for 2 min, and relative light units (RLU) were observed with an Ultra luminometer.

### Spleen and lymph node immunophenotyping by FCM

Immunized mice were sacrificed 7 days after the second booster immunization, and spleens and lymph nodes were collected. Spleen samples within lymphocyte separation medium were mashed through 70 μm cell strainers to harvest single cell suspensions (DKW33-R0100), and the suspension was centrifuged according to the manufacturer’s instructions. The cell suspension was cleaned with PBS, processed with lysis buffer at 25 °C to lyse erythrocytes, and resuspended in PBS supplemented with 1% BSA after termination of lysis. Lymph node samples were minced using a pestle and mashed through a 70 μm cell strainer within PBS. Single cell suspensions were incubated with the following antibodies to GC B and Tfh cell antigens for 30 min at 4 °C: anti-mouse CD3 (PerCP/Cyanine5.5), anti-mouse CD4 (FITC), anti-mouse CD19 (PE), anti-mouse PD-1 (Brilliant Violet 421), anti-mouse CXCR5 (APC), anti-mouse CD19 (Brilliant Violet 421), anti-mouse GL7 (FITC), and anti-mouse CD95 (PE-CF594).

### Splenocyte restimulation assay

Splenocyte suspension, obtained from previous section, was washed with PBS, resuspended in RPMI 1640 involving100 U/mL IL-2 (Novoprotein, China), 1 mM sodium pyruvate (Sigma-Aldrich, USA), and 0.05 mM β-mercaptoethanol (Sigma-Aldrich, USA), and plated in 12-well plates (1 × 10^6^ cells/well). RBD_β_-HR/trimer protein was added to12-well plates at a concentration of 10 μg/mL. After incubating for 3 days at 37 °C, the cells were collected for FCM, and the supernatant was harvested and stored at -80 °C until cytokine assay.

### Statistical analysis

Statistical analysis was processed with GraphPad Prism 8.0 (GraphPad Software). Statistical values were analyzed by One-way analysis of variance (ANOVA). *p* value < 0.05 were statistically significant (*p < 0.05; **p < 0.01; ***p < 0.001; ****p < 0.0001).

## Data Availability

The data primarily presented in the current study are available from the corresponding author upon reasonable request.

## References

[1] He C, Yang J, He X, Hong W, Lei H, Chen Z (2021). A bivalent recombinant vaccine targeting the S1 protein induces neutralizing antibodies against both SARS-CoV-2 variants and wild-type of the virus. MedComm (2020).

[2] Shereen MA, Khan S, Kazmi A, Bashir N, Siddique R (2020). COVID-19 infection: Origin, transmission, and characteristics of human coronaviruses. J Adv Res.

[3] Hoffmann M, Kleine-Weber H, Schroeder S, Kruger N, Herrler T, Erichsen S (2020). SARS-CoV-2 cell entry depends on ACE2 and TMPRSS2 and is blocked by a clinically proven protease inhibitor. Cell.

[4] Cui J, Li F, Shi ZL (2019). Origin and evolution of pathogenic coronaviruses. Nat Rev Microbiol.

[5] Tuekprakhon A, Nutalai R, Dijokaite-Guraliuc A, Zhou D, Ginn HM, Selvaraj M (2022). Antibody escape of SARS-CoV-2 Omicron BA.4 and BA.5 from vaccine and BA.1 serum. Cell.

[6] Desingu PA, Nagarajan K, Dhama K (2022). Emergence of Omicron third lineage BA.3 and its importance. J Med Virol.

[7] Wang P, Liu L, Iketani S, Luo Y, Guo Y, Wang M, et al. Increased Resistance of SARS-CoV-2 Variants B.1.351 and B.1.1.7 to Antibody Neutralization. Res Sq. 2021. 10.21203/rs.3.rs-155394/v1.

[8] Servellita V, Morris MK, Sotomayor-Gonzalez A, Gliwa AS, Torres E, Brazer N (2022). Predominance of antibody-resistant SARS-CoV-2 variants in vaccine breakthrough cases from the San Francisco Bay Area. California. Nat Microbiol.

[9] Tegally H, Moir M, Everatt J, Giovanetti M, Scheepers C, Wilkinson E (2022). Emergence of SARS-CoV-2 Omicron lineages BA.4 and BA.5 in South Africa. Nat Med.

[10] Wilhelm A, Widera M, Grikscheit K, Toptan T, Schenk B, Pallas C (2022). Limited neutralisation of the SARS-CoV-2 Omicron subvariants BA.1 and BA.2 by convalescent and vaccine serum and monoclonal antibodies. EBioMedicine.

[11] Pfizer and BioNTech provide update on Omicron variant.

[12] Cameroni E, Bowen JE, Rosen LE, Saliba C, Zepeda SK, Culap K, et al. Broadly neutralizing antibodies overcome SARS-CoV-2 Omicron antigenic shift. Nature. 2022;602(7898):664-70. 10.1038/s41586-021-04386-2. 10.1038/s41586-021-04386-2 PMC9531318 35016195

[13] Planas D, Saunders N, Maes P, Guivel-Benhassine F, Planchais C, Buchrieser J (2022). Considerable escape of SARS-CoV-2 Omicron to antibody neutralization. Nature.

[14] Callaway E (2021). Heavily mutated Omicron variant puts scientists on alert. Nature.

[15] Tubiana J, Xiang Y, Fan L, Wolfson HJ, Chen K, Schneidman-Duhovny D, et al. Reduced antigenicity of Omicron lowers host serologic response. bioRxiv. 2022 10.1101/2022.02.15.480546 10.1016/j.celrep.2022.111512 PMC9515332 36223774

[16] Sun S, Cai Y, Song TZ, Pu Y, Cheng L, Xu H (2021). Interferon-armed RBD dimer enhances the immunogenicity of RBD for sterilizing immunity against SARS-CoV-2. Cell Res.

[17] Dai L, Gao GF (2021). Viral targets for vaccines against COVID-19. Nat Rev Immunol.

[18] Ma X, Zou F, Yu F, Li R, Yuan Y, Zhang Y (2020). Nanoparticle vaccines based on the receptor binding domain (RBD) and heptad repeat (HR) of SARS-CoV-2 elicit robust protective immune responses. Immunity.

[19] https://covariants.org. Accessed on: 2022/10/30.

[20] Wang GL, Wang ZY, Duan LJ, Meng QC, Jiang MD, Cao J (2021). Susceptibility of circulating SARS-CoV-2 variants to neutralization. N Engl J Med.

[21] Henao-Tamayo MI, Ordway DJ, Irwin SM, Shang S, Shanley C, Orme IM (2010). Phenotypic definition of effector and memory T-lymphocyte subsets in mice chronically infected with Mycobacterium tuberculosis. Clin Vaccine Immunol.

[22] Zaph C, Uzonna J, Beverley SM, Scott P (2004). Central memory T cells mediate long-term immunity to Leishmania major in the absence of persistent parasites. Nat Med.

[23] Stebegg M, Kumar SD, Silva-Cayetano A, Fonseca VR, Linterman MA, Graca L (2018). Regulation of the germinal center response. Front Immunol.

[24] Robinson C, Baehr C, Schmiel SE, Accetturo C, Mueller DL, Pravetoni M (2019). Alum adjuvant is more effective than MF59 at prompting early germinal center formation in response to peptide-protein conjugates and enhancing efficacy of a vaccine against opioid use disorders. Hum Vacc Immunother.

[25] Crotty S (2019). T follicular helper cell biology: a decade of discovery and diseases. Immunity.

[26] Laidlaw BJ, Ellebedy AH (2022). The germinal centre B cell response to SARS-CoV-2. Nat Rev Immunol.

[27] Cavazzoni CB, Hanson BL, Podesta MA, Bechu ED, Clement RL, Zhang HC, et al. Follicular T cells optimize the germinal center response to SARS-CoV-2 protein vaccination in mice. Cell Rep. 2022;38(8):110399. 10.1016/j.celrep.2022.110399. 10.1016/j.celrep.2022.110399 PMC8806144 35139367

[28] Gattinger P, Tulaeva I, Borochova K, Kratzer B, Trapin D, Kropfmuller A, et al. Omicron: A SARS-CoV-2 variant of real concern. Allergy. 2022;77(5):1616-20. https://onlinelibrary.wiley.com/doi/10.1111/all.15264. 10.1111/all.15264 PMC9111213 35188670

[29] Ravichandran S, Coyle EM, Klenow L, Tang J, Grubbs G, Liu S (2020). Antibody signature induced by SARS-CoV-2 spike protein immunogens in rabbits. Sci Transl Med.

[30] Dai L, Zheng T, Xu K, Han Y, Xu L, Huang E (2020). A universal design of Betacoronavirus vaccines against COVID-19, MERS, and SARS. Cell.

[31] He C, Yang J, Hong W, Chen Z, Peng D, Lei H (2022). A self-assembled trimeric protein vaccine induces protective immunity against Omicron variant. Nat Commun.

[32] Yang J, Wang W, Chen Z, Lu S, Yang F, Bi Z (2020). A vaccine targeting the RBD of the S protein of SARS-CoV-2 induces protective immunity. Nature.

[33] van Oosten L, Altenburg JJ, Fougeroux C, Geertsema C, van den End F, Evers WAC (2021). Two-component nanoparticle vaccine displaying glycosylated Spike S1 domain induces neutralizing antibody response against SARS-CoV-2 variants. mBio.

[34] Tian JH, Patel N, Haupt R, Zhou H, Weston S, Hammond H (2021). SARS-CoV-2 spike glycoprotein vaccine candidate NVX-CoV2373 immunogenicity in baboons and protection in mice. Nat Commun.

[35] Mi Y, Xie T, Zhu B, Tan J, Li X, Luo Y (2021). Production of SARS-CoV-2 virus-like particles in insect cells. Vaccines (Basel).

[36] Felberbaum RS (2015). The baculovirus expression vector system: A commercial manufacturing platform for viral vaccines and gene therapy vectors. Biotechnol J.

[37] Pollet J, Chen WH, Strych U (2021). Recombinant protein vaccines, a proven approach against coronavirus pandemics. Adv Drug Deliv Rev.

[38] Jeyanathan M, Afkhami S, Smaill F, Miller MS, Lichty BD, Xing Z (2020). Immunological considerations for COVID-19 vaccine strategies. Nat Rev Immunol.

[39] Sathe A, Cusick JK. Biochemistry, immunoglobulin M. StatPearls. 2022. 32310455

[40] Steinbuck MP, Seenappa LM, Jakubowski A, McNeil LK, Haqq CM, DeMuth PC (2021). A lymph node-targeted Amphiphile vaccine induces potent cellular and humoral immunity to SARS-CoV-2. Sci Adv.

[41] Garcia-Beltran WF, Lam EC, Astudillo MG, Yang D, Miller TE, Feldman J (2021). COVID-19-neutralizing antibodies predict disease severity and survival. Cell.

[42] Doria-Rose NA, Shen X, Schmidt SD, O'Dell S, McDanal C, Feng W, et al. Booster of mRNA-1273 strengthens SARS-CoV-2 Omicron neutralization. medRxiv. 202110.1101/2021.12.15.21267805

[43] Tada T, Zhou H, Samanovic MI, Dcosta BM, Cornelius A, Mulligan MJ, et al. Comparison of neutralizing antibody titers elicited by mRNA and adenoviral vector vaccine against SARS-CoV-2 variants. bioRxiv. 202110.1101/2021.07.19.452771

[44] Li Z, Xiang T, Liang B, Deng H, Wang H, Feng X (2021). Characterization of SARS-CoV-2-specific humoral and cellular immune responses induced by inactivated COVID-19 vaccines in a real-world setting. Front Immunol.

[45] Carmen JM, Shrivastava S, Lu Z, Anderson A, Morrison EB, Sankhala RS (2021). SARS-CoV-2 ferritin nanoparticle vaccine induces robust innate immune activity driving polyfunctional spike-specific T cell responses. NPJ Vaccines.

[46] Nanishi E, Borriello F, O'Meara TR, McGrath ME, Saito Y, Haupt RE (2022). An aluminum hydroxide:CpG adjuvant enhances protection elicited by a SARS-CoV-2 receptor binding domain vaccine in aged mice. Sci Transl Med.

[47] Baumjohann D, Fazilleau N (2021). Antigen-dependent multistep differentiation of T follicular helper cells and its role in SARS-CoV-2 infection and vaccination. Eur J Immunol.

[48] Lederer K, Castano D, Atria DG, Oguin TH, Wang S, Manzoni TB (2020). SARS-CoV-2 mRNA vaccines foster potent antigen-specific germinal center responses associated with neutralizing antibody generation. Immunity.

[49] Ko EJ, Kang SM (2018). Immunology and efficacy of MF59-adjuvanted vaccines. Hum Vaccin Immunother.

